# Effectiveness and mechanisms of adipose-derived stem cell therapy in animal models of Parkinson’s disease: a systematic review and meta-analysis

**DOI:** 10.1186/s40035-021-00238-1

**Published:** 2021-04-29

**Authors:** Keya Li, Xinyue Li, Guiying Shi, Xuepei Lei, Yiying Huang, Lin Bai, Chuan Qin

**Affiliations:** NHC Key Laboratory of Human Disease Comparative Medicine, Beijing Key Laboratory for Animal Models of Emerging and Reemerging Infectious Diseases, Institute of Laboratory Animal Science, Chinese Academy of Medical Sciences and Comparative Medicine Center, Peking Union Medical College, Beijing, 100021 China

**Keywords:** Parkinson’s disease, Adipose-derived stem cells, Animal model, Stem cell therapy, Meta-analysis, Neurogenesis, Neuroprotection

## Abstract

Animal models provide an opportunity to assess the optimal treatment way and the underlying mechanisms of direct clinical application of adipose-derived stem cells (ADSCs). Previous studies have evaluated the effects of primitive and induced ADSCs in animal models of Parkinson’s disease (PD). Here, eight databases were systematically searched for studies on the effects and in vivo changes caused by ADSC intervention. Quality assessment was conducted using a 10-item risk of bias tool. For the subsequent meta-analysis, study characteristics were extracted and effect sizes were computed. Ten out of 2324 published articles (*n* = 169 animals) were selected for further meta-analysis. After ADSC therapy, the rotation behavior (10 experiments, *n* = 156 animals) and rotarod performance (3 experiments, *n* = 54 animals) were improved (*P* < 0.000 01 and *P* = 0.000 3, respectively). The rotation behavior test reflected functional recovery, which may be due to the neurogenesis from neuronally differentiated ADSCs, resulting in a higher pooled effect size of standard mean difference (SMD) (− 2.59; 95% CI, − 3.57 to − 1.61) when compared to that of primitive cells (− 2.18; 95% CI, − 3.29 to − 1.07). Stratified analyses by different time intervals indicated that ADSC intervention exhibited a long-term effect. Following the transplantation of ADSCs, tyrosine hydroxylase-positive neurons recovered in the lesion area with pooled SMD of 13.36 [6.85, 19.86]. Transplantation of ADSCs is a therapeutic option that shows long-lasting effects in animal models of PD. The potential mechanisms of ADSCs involve neurogenesis and neuroprotective effects. The standardized induction of neural form of transplanted ADSCs can lead to a future application in clinical practice.

**Supplementary Information:**

The online version contains supplementary material available at 10.1186/s40035-021-00238-1.

## Introduction

Parkinson’s disease (PD) is the second most common progressive neurodegenerative disorder. It was initially described as the ‘shaking palsy’ by James Parkinson in 1877 [1]. Pathologically, PD is characterized by a prominent loss of midbrain dopamine (DA)-secreting neurons with the presence of α-synuclein-containing Lewy bodies, which result in a series of clinical features [1]. The clinical symptoms of PD include non-motor and motor symptoms such as rigidity, bradykinesia, and essential resting tremor, which is distinct from the tremors caused by epilepsy, alcoholism, or stroke [2, 3]. It has been reported that male adults over 50 years of age are susceptible to PD [4]. With the increased aging population, disability and death from neurological disorders have been on the rise [5]. PD has now become the leading cause of disability worldwide. In 2016, it was reported that PD had caused 3,200,000 disability-adjusted of life years and more than 20,000 mortalities [4].

Current therapeutic options for PD are aimed at improving the motor symptomatology in PD patients using pharmacologic agents [6, 7]. However, DA agonists can cause side effects such as impulse control disorders, which has limited their long-term application [7]. Although medical treatment has been performed in PD patients since the early 1960s [1], the disease remains incurable so far. Even in early stages of PD, surgical techniques can only control and retard the progression of PD. Therefore, it is important to develop novel therapeutic strategies for the treatment of PD. Since the progression of PD involves DA neuron loss in the substantia nigra (SN), dopaminergic protection and cell replacement have been proposed as critical perspectives for curing this disease.

Mesenchymal stem cells (MSCs) are adult stem cells that have immunosuppressive functions and are less prone to tumorigenesis. Unlike embryonic or fetal-derived cell transplantation, the transplantation of autologous adult stem cells is not hindered by ethical issues or safety limitations [6, 8]. Adipose-derived stem cells (ADSCs) are a primary source of MSCs. They are abundant and easy to be isolated through less invasive procedures compared to the bone marrow MSCs. They have a great proliferative potential, can be expanded through multiple passages in vitro with minimal senescence, and offer direct application in the field of tissue engineering [9–11]. They can also be selectively differentiated into endoderm and ectoderm cell lineages in vitro under appropriate conditions [12–15].

With these advantages, ADSCs have been evaluated for the treatment of different diseases. They have been intra-articularly administered for the treatment of osteoarthritis [16], percutaneously as well as endoscopically injected for the management of postoperative enterocutaneous fistula [17], and intracerebroventricularly injected in human brains for neurodegenerative diseases [18].

Animal models of PD are often established with 6-hydroxydopamine (6-OHDA) [19–27], rotenone [28, 29], 1-methyl-4-phenyl-1,2,3,6-tetrahydropyridine (MPTP) [30, 31] and lipopolysaccharide [32]. The animal models have been used to study the therapeutic effects of ADSCs for PD, with varied transplantation forms and follow-up times.

In this review, we perform meta-analysis of preclinical studies to estimate the optimal transplantation route and therapeutic effects of ADSCs in PD animal models, and summarize the therapeutic mechanisms of ADSCs.

## Methods

### Search methods

Online search was performed in eight databases (PubMed, Embase, Web of Science, Scopus, CINAHL, Cochrane Library, Medline, ProQuest Dissertations and Theses) to identify studies on the effects and mechanisms of ADSC treatment in animal models of PD by the date of March 21, 2020. The PICO (population, interventions, comparators, and outcomes) strategy was used as a reminder of the scope of the review, defined by the types of population (participants), types of interventions (and comparisons), and the types of outcomes that are of interest [33]. The systematic search in PubMed is described in Supplementary file 1.

### Inclusion criteria

The inclusion criteria are described in Table S1 using the PICO strategy. All the included articles should be in English and provide experimental data. Briefly, we selected peer-reviewed studies on the effects and *in vivo* changes caused by ADSC interventions in common models of PD. Experiments should have been prospectively controlled, and behavioral outcomes and *in vivo* data after transplantation should have been provided. Titles were evaluated for possible duplicates, type of article (not a review), type of research (animal studies), and theme fitted. Abstracts were reviewed for experimental PD models and ADSC intervention. Full-text papers were assessed for study design, treatment and control groups, and outcome measurements.

### Outcome measurements

Studies reporting the following outcomes were selected: behavioral tests like rotation (turns/min) or rotarod (s) tests, and *in vivo* changes like DA neurons, protein expression after transplantation or positron emission tomography/magnetic resonance imaging.

### Risk of bias of included studies

Study quality and risk of bias were assessed by the SYRCLE's risk of bias (RoB) tool provided by the SYstematic Review Centre for Laboratory Animal Experimentation. This tool, based on the Cochrane Collaboration RoB Tool, aims to assess the methodological quality and has been adapted to aspects of bias that play a role in animal experiments [34]. This tool contains 10 items that are associated with 6 types of bias: selection bias, performance bias, detection bias, attrition bias, reporting bias, and other biases. The 10 items are organized into subitems in the form of questions with an answer of “Yes”, “No” or “Unclear”. “Yes” indicates a bias with low risk, “No” refers to a high risk of bias, while “Unclear” means that the risk of bias is unknown, usually because that the item was not reported. The assessment was performed by two reviewers independently, and any controversy was resolved by discussion. An overview of this RoB tool entry is shown in Table S2.

### Data extraction and management

The following information was extracted from each article: author information, publication year, source of ADSCs, animal species, lesion models, number of animals per study arm group, administration route, ADSC doses, number of days between lesioning and treatment, follow-up duration, and the outcome data, including rotation behavior, rotarod test results, and tyrosine hydroxylase (TH)-positive neurons. Data in text, tables and graphs were extracted. Studies that mentioned both primitive ADSCs and neural-induction ADSCs were considered as two independent experiments. They were discerned into subgroups according to the type of intervention: Primitive ADSCs and neural induction form of ADSCs, and according to the valid duration: follow-up time ≤4 weeks; and follow-up time >4 weeks. We only extracted behavioral data at the final time point of serial behavioral testing. Where the outcomes were reported graphically but not as numerical data in the text, values of mean and standard deviation (SD) or standard error of the mean (SEM) were extracted from the images by two authors (KYL and XYL) using WebPlotDigitizer 4.2. Readings by them were averaged and SEM was converted to SD for data analysis.

### Statistical analysis

Treatment effects are expressed as standard mean difference (SMD) for continuous variables, for comparison of ADSC *versus* control groups. We applied the DerSimonian and Laird random-effects model for anticipated heterogeneity of the extracted data [35]. SMD was calculated with the Hedges statistical method and is displayed with 95% confidence interval (95% CI) in forest plots. Heterogeneity among studies was assessed using the *I*^2^ statistics, and the statistical significance of the pooled effect size among studies was determined by the z-test. To determine whether our findings were highly influenced by any single study, sensitivity analysis was performed by iteratively removing one study at a time. Moreover, potential publication bias was evaluated by Egger’s and Begg’s tests and displayed in funnel plots, in which a publication bias was considered when *P* < 0.05. All analyses were performed with Review Manager 5.3 for Mac (Copenhagen: The Nordic Cochrane Centre, The Cochrane Collaboration) and Stata/SE 15.0 for Mac (StataCorp LP, College Station, TX).

## Results

### Characteristics of the included studies

**Fig. 1:**
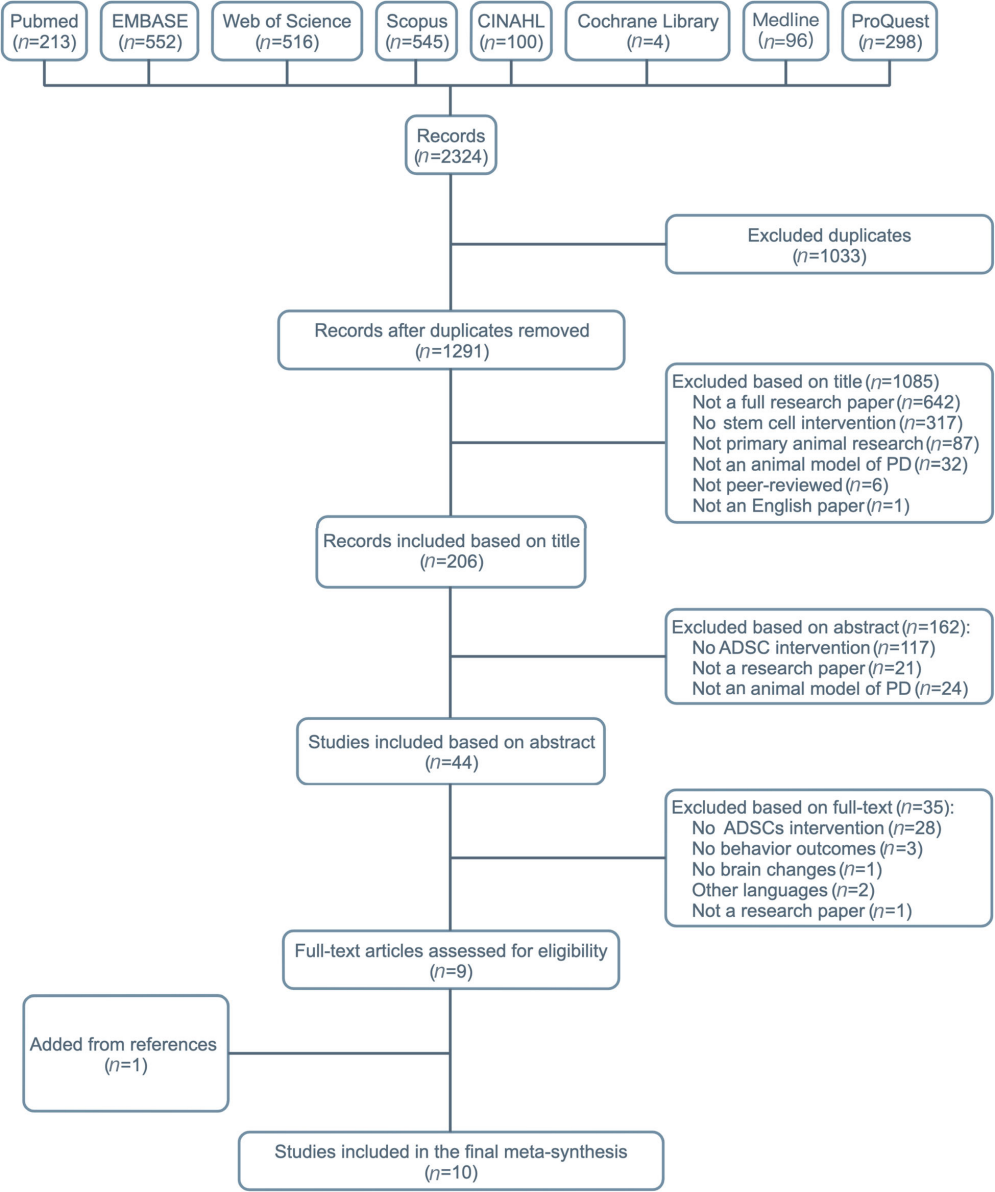
Flow chart of study selection

A total of 2,324 studies were identified from the systematic search, among which 10 studies [19, 20, 22–25, 27, 28, 30, 31] met the inclusion criteria after full-text assessment (Fig. 1, Table 1 and Table S3).

**Table 1:** List of all 10 studies that met the inclusion criteria based on full-text assessment

Author (year)	Type of intervention	Model	Species/Strains	Route of administration	Doses per animal	Follow-up time	Outcome parameters
McCoy (2008) [19]	Neural induction	6-OHDA	Rats/Sprague-Dawley	Intrastriatal	4 × 10^4^	4 weeks	Rotation (turns/20 min) Number of TH^+^ neurons in SNpc and striatum Microglial burden in SNpc (IOD)
Zhou (2013) [30]	Neural induction	MPTP (hemi)	Monkeys/Rhesus	Intrastriatal	6 × 10^6^	4 months	UPDRS (scores) Rotation (turns/min) Percentage of TH^+^ neurons in the substantia nigra
Park (2014) [20]	Original	6-OHDA	Rats/Sprague-Dawley	Cisterna magna injection	2 × 10^6^	6 weeks	Rotation (turns/50 min) Relative neuronal protein expression of midbrain tissues
Berg (2015) [24]	Original	6-OHDA	Rats/Wistar	Intranigral	3 × 10^5^	3 weeks	8-arm radial maze (times) Rotation (turns/min) Number of newly generated cells in the adult DG Nigral mRNA levels of GDNF, BDNF and GFAP Microglia number in the transplantation area
Choi (2015) [23]	Original	6-OHDA	Mice/C57BL6	Intravenous	1 × 10^6^	6 weeks	Rotation (turns/30 min) Rotarod (s) Number of TH Neurons in the SN PET imaging analysis of dopamine D2 receptor in the striatum (binding potential) The population of damaged mitochondria (%) Mitochondrial complex I activity (%)
Schwerk (2015) [22]	Original	6-OHDA	Rats/Wistar	Intranigral	3 × 10^5^	6 months	Rotation (turns/min) 8-arm radial maze (times) Percentage of TH^+^ neurons in the SN (%) Neurogenesis of subventricular and hippocampal cells (cell number) EPO, IL-10, IL-4, and IL-2 levels (pg/ml)
Takahashi (2017) [25]	Neural induction	6-OHDA	Rats/Wistar	Ipsilateral MFB injection	4 × 10^5^	4 weeks	Rotation (turns/min)
Chi (2018) [31]	Original	MPTP	Mice/C57BL6	Intrastriatal	1 × 10^6^	3 weeks	Beam walking (s) Rotarod (s) Locomotor activity Comparable TH quantity of the SN (%)
Meligy (2019) [28]	Original	Rotenone	Rats/Wistar	Intracardiac	1 × 10^6^	2 weeks	Rotarod (s) Activity cage (counts/5 min) Pole test (s) Pale and dark neurons in SNc (cell number) TH^+^ cells in the SNc (cells/mm^2^) Blood levels of angiopoietin-2 (ng/ml) and dopamine (pg/ml) GFAP and Nestin mRNA gene expression
Moayeri (2020) [27]	Original	6-HD	Rats/Sprague-Dawley	Ipsilateral MFB injection	3 × 10^5^	6 weeks	Rotation (turns/1 h) Nissl-stained cells in the SNc

*Abbreviations*: *TH* Tyrosine hydroxylase, *SNc* substantia nigra compacta, *DG* dentate gyrus, *MFB* medial forebrain bundle, *BP n*-butylidenephthalid

The 10 studies involved 169 animals (control group, *n* = 74; treatment group, *n* = 95). The most frequently used PD model was the 6-OHDA model (7 studies), followed by the MPTP (2 studies) and rotenone (1 study) models. Rats were the most preferred animals for experimentation (Table S4).

The effectiveness of ADSC intervention included decreased rotation numbers and longer stay on the rotarod (expressed in SMD). Histologically, TH-positive neurons in 3 experiments from 3 studies were scored in uniform scoring scales. Therefore, meta-analyses were performed on these outcomes.

### Assessment of the risk of bias

**Fig. 2:**
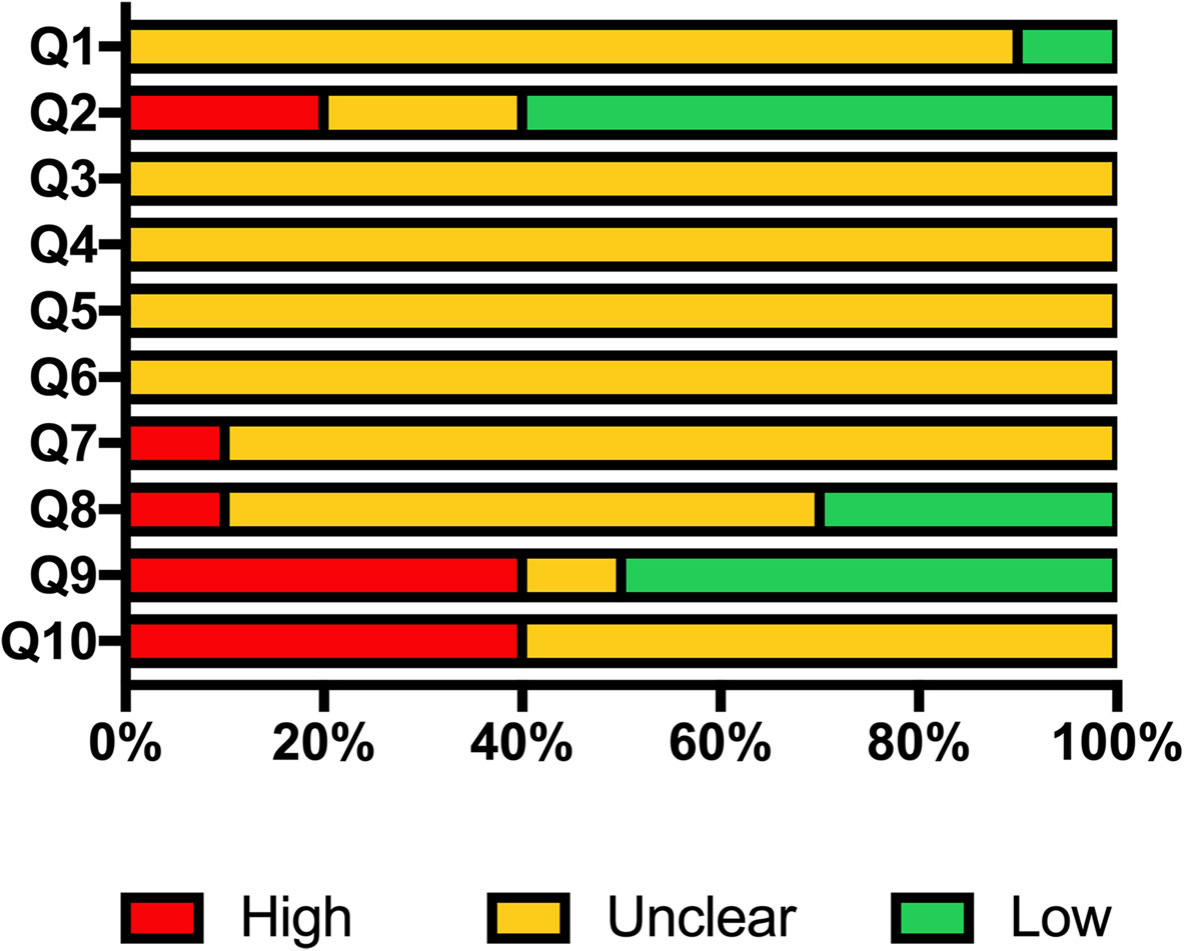
Quality assessment of the 10 studies. Q1. Was the allocation sequence adequately generated and applied? Q2. Were the groups similar at baseline or were they adjusted for confounders in the analysis? Q3. Was the allocation to the different groups adequately concealed during the experiment? Q4. Were the animals randomly housed during the experiment? Q5. Were the caregivers and/or investigators blinded from knowledge which intervention each animal received during the experiment? Q6. Were animals selected at random for outcome assessment? Q7. Was the outcome assessor blinded? Q8. Were incomplete outcome data adequately addressed? Q9. Are reports of the study free of selective outcome reporting? Q10. Was the study apparently free of other problems that could result in high risk of bias?

The risk-of-bias summary of each study based on the SYRCLE’s RoB tool is shown in Table S5, and the main observation was “unclear” (Fig. 2). As for the selection bias (Q1–Q3), animals were randomly assigned in only one study (10%; Q1); baseline similarities were more often in these articles (60%; Q2), whereas no studies documented the information regarding allocation concealment (Q3). Performance bias could not be established (Q4 and Q5). None of the articles reported random housing, although it is unlikely that outcomes were influenced by lack of random housing (Q4). There was no blinding of caregivers and/or investigators (Q5). With respect to detection bias (Q6 and Q7), none of the studies randomly selected animals for outcome assessment (Q6). In addition, the outcome assessor in one study was not blinded (10%; Q7). However, the outcome was not likely to be impacted by lack of blinding (Q7). Incomplete outcome data were addressed in one study (10%; Q8), and a low risk was identified in 3 studies. With respect to the reporting bias (Q9) and other bias (Q10), high risks were identified in 4 studies (40%). The high risks in item 10 were exclusively due to a lack of specified SD or SEM.

### Therapeutic effects of ADSCs

There was an average of 0.32% difference in the rotation and rotarod test data extracted by the two authors.

#### Rotation behavior

**Fig. 3:**
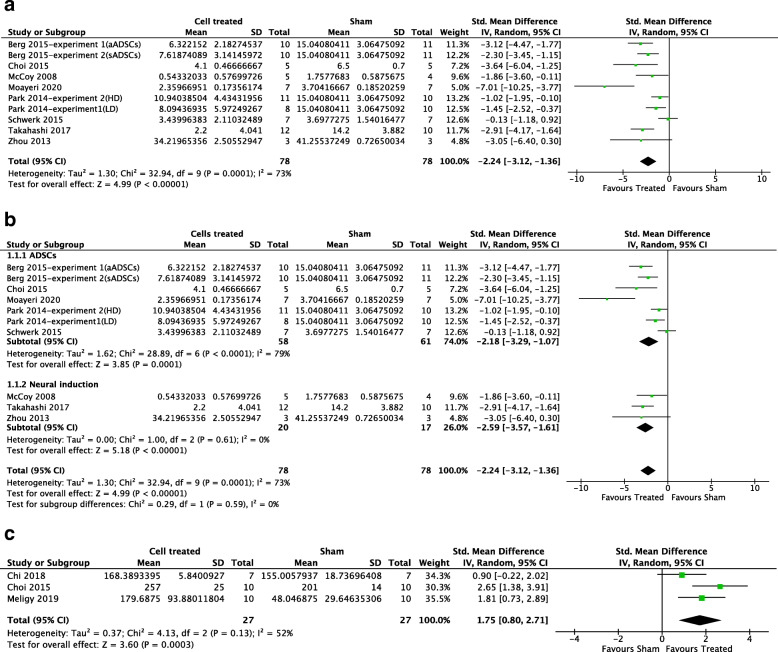
Effects of ADSCs intervention. **a** Effect of ADSC intervention on rotation behavior reported in 10 individual experiments. The pooled effect size of standard mean difference (SMD) for rotation improvement was − 2.24 (95% CI, − 3.12 to − 1.36, z-value = 3.85, *P* < 0.000 01). **b** Stratified meta-analysis of the effects of ADSC intervention on rotation behavior by the type of ADSCs (original ADSCs, pooled estimate − 2.18, 95% CI − 3.29 to − 1.07, *P* = 0.000 1; and neural induction form of ADSCs, pooled estimate − 2.59, 95% CI − 3.57 to − 1.61, *P* < 0.000 01). **c** Effect of ADSC intervention on rotarod test reported in 3 individual experiments. The pooled effect size of SMD was 1.75 (95% CI, 0.80 to 2.71, z-value = 3.60, *P* = 0.000 3)

Ten experiments from 8 studies involving 165 animals tested the effects of ADSCs on rotation behavior, and showed the same direction of effect. The pooled effect size of SMD for ADSC efficacy in PD treatment was −2.24 (95% CI, −3.12 to −1.36, z = 4.99, *P* < 0.000 01), which demonstrated substantial and significant attenuation of apomorphine/amphetamine-induced rotational behavior by ADSC treatment (Fig. 3a). Both primitive ADSCs (7 experiments) and neural-induction ADSCs (3 experiments) contributed to behavioral improvement (*P* < 0.000 01) (Fig. 3b). The neural-induction ADSCs exhibited a more synergic effect size (SMD, −2.59; 95% CI, −3.57 to −1.61) than the primitive ones (SMD, −2.18; 95% CI, −3.29 to −1.07).

#### Rotarod test

Three experimental studies (including 54 animals) evaluated the effects of ADSCs on rotarod test performance. The results showed that ADSC intervention could significantly prolong the latency to fall during the rotarod test of PD models (overall pooled SMD, 1.75; 95% CI, 0.80–2.71; z = 3.60; *P* = 0.000 3) (Fig. 3c).

### Subgroup analysis

**Fig. 4:**
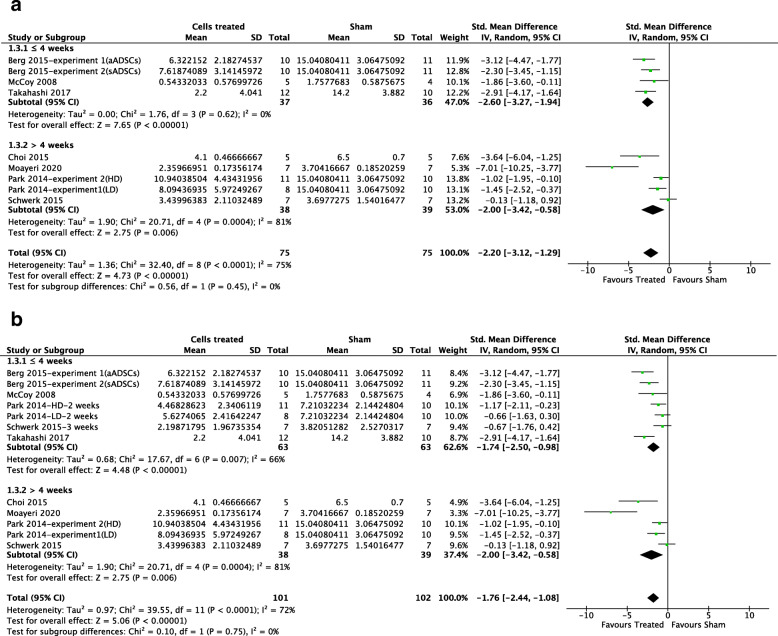
Subgroup analyses by ADSC intervention period. **a** Effect of ADSC intervention period on rodent rotation behavior reported in 9 individual experiments. The pooled effect size of SMD for the subgroup of recording time point ≤4 weeks was − 2.60 (95% CI, − 3.27 to − 1.94, z-value = 7.65, *P* < 0.000 01). **b** Data at shorter follow-up but with most improved effects from the > 4 weeks subgroup were added to the ≤4 weeks subgroup. The pooled effect size of SMD was − 1.74 (95% CI, − 2.50 to − 0.98, z-value = 4.48, *P* < 0.000 01). The pooled effect size of SMD for rotation improvement at follow-up time over 4 weeks was − 2.00 (95% CI, − 3.42 to − 0.58, z-value = 2.75, *P* = 0.006)

Since the follow-up time may impact the intervention effects, the studies were divided into two groups according to the follow-up time. In this analysis, the only one study with a monkey model was excluded. Analysis of data at the final point of follow-up showed that the improvement of rotation behavior weakened over time (Fig. 4a). The overall-effect z value of the ≤ 4 weeks subgroup was 7.65 (*P* < 0.000 01) and the pooled SMD was −2.60 (95% CI, −3.27 to −1.94), while the overall-effect z value and pooled SMD of the > 4 weeks subgroup were 2.75 (*P* = 0.006) and −2.00 (95% CI, −3.42 to −0.58), respectively. In fact, in the studies with follow-up time over 4 weeks, the rotation behavior of animals had been improved before the final time point. Considering the between-group differences, we included data at shorter follow-up but with most improved effects from the > 4 weeks subgroup to the ≤ 4 weeks subgroup (Fig. 4b). The overall-effect z-value of the ≤ 4 weeks subgroup was 4.48 (*P* < 0.000 01) while the pooled SMD with 95% CI was −1.74[−2.50, −0.98]. These results suggest that ADSC intervention exerts a long-term effect.

### Mechanisms of the therapeutic effect

#### Neurogenesis

**Fig. 5:**
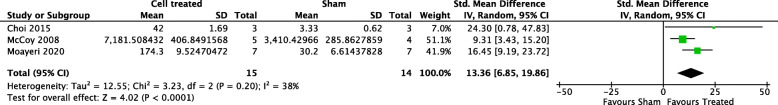
Meta-analysis of the effects of ADSC intervention on TH^+^ neurons in the SN. The effect of ADSC intervention on dopaminergic neuron generation was reported in 7 individual experiments and 3 of them met the criteria for meta-analysis. The pooled effect size of SMD was 13.36 (95% CI 6.85 to 19.86, z-value = 4.02, *P* < 0.000 1)

All of the 10 studies have mentioned DA neuron survival after ADSC intervention in PD models. Seven studies [19, 22–24, 27, 28, 31] presented quantitative analysis of TH-positive neurons in the brain, which consistently showed that the DA neurons recovered after ADSC transplantation. Three of them [19, 23, 27] evaluated TH^+^ neurons in the SN, so the numbers of TH^+^ neurons were combined for meta-analysis. Results showed that the overall pooled SMD was 13.36 (95% CI, 6.85–19.86) and the z-value was 4.02 (*P* < 0.000 01), favoring the use of ADSCs over controls for the outcome of TH^+^ neuron numbers within the lesioned side (Fig. 5). Heterogeneity was not significant (*I*^*2*^ = 38%) [36].

Therapeutic ADSCs have also been shown to increase striatum TH^+^ neurons [19], hippocampal dentate gyrus cells [24], and neurogenesis in the subventricular region with long-term increased expression of proliferating cell nuclear antigen in the ipsilateral midbrain, a marker for cell proliferation [22]. Six months after transplantation, there were significantly more BrdU+ cells in the subgranular zone of the hippocampus when compared to the 6-OHDA model with control treatment [22]. Park JB et al. have documented that the PI3K/Akt pathway is involved in neuronal growth promotion by ADSCs cultured at high cell density [20].

### Neuroprotection

In addition to the region-specific neurogenesis, primitive ADSCs can generate a broad range of neuroprotective effects.

McCoy has reported that microglial activation markers are decreased in the SN after intrastriatal injection of ADSCs, indicating a reduction in microglial density [19]. Erythropoietin, which plays antiapoptotic, antioxidant and anti-inflammatory roles in neurons [37, 38], has been found to be increased after transplantation [22].

Neurotrophic factors affect neuronal functions. They have the potential to protect DA neurons from degeneration and promote regeneration of the nigrostriatal DA system. Accumulating evidence has shown increased expression of brain-derived neurotrophic factor (BDNF) after ADSC transplantation *in vitro* and *in vivo*. The induction of interferons (IFNs) by ADSCs promotes the synthesis of BDNF through the IFN-β signaling [20]. Moreover, ADSCs have been found to produce nerve growth factor, glial-derived neurotrophic factor [20, 22, 24], vascular endothelial growth factor, von Willebrand factor, basic fibroblast growth factor and insulin-like growth factor-1. These findings suggest the role of ADSCs in the induction of angiogenesis [20, 28].

In addition, ADSCs express high levels of interleukin 10 (IL-10), IL-2 and IL-4, and restore mitochondrial dysfunction as well as the mitochondrial complex I activity, which is known to be inhibited by 6-OHDA [23].

In summary, therapeutic ADSCs exert their neuroprotective effects through neuroinflammation attenuation, immunoregulation, secretion of neurotropic and growth factors, recovery of dysfunctional mitochondria, and microenvironmental protection.

### Sensitivity

Sensitivity analysis is used to detect whether the pooled results are still stable for their potential impact. We performed a leave-one-out sensitivity analysis by iteratively removing one study at a time and recalculating the pooled effect size of the remaining studies. For rotation behavior, rotarod test result and TH-positive neurons, the pooled effect was stable, indicating that the meta-analysis results were not driven by any single study.

### Publication bias

**Fig. 6:**
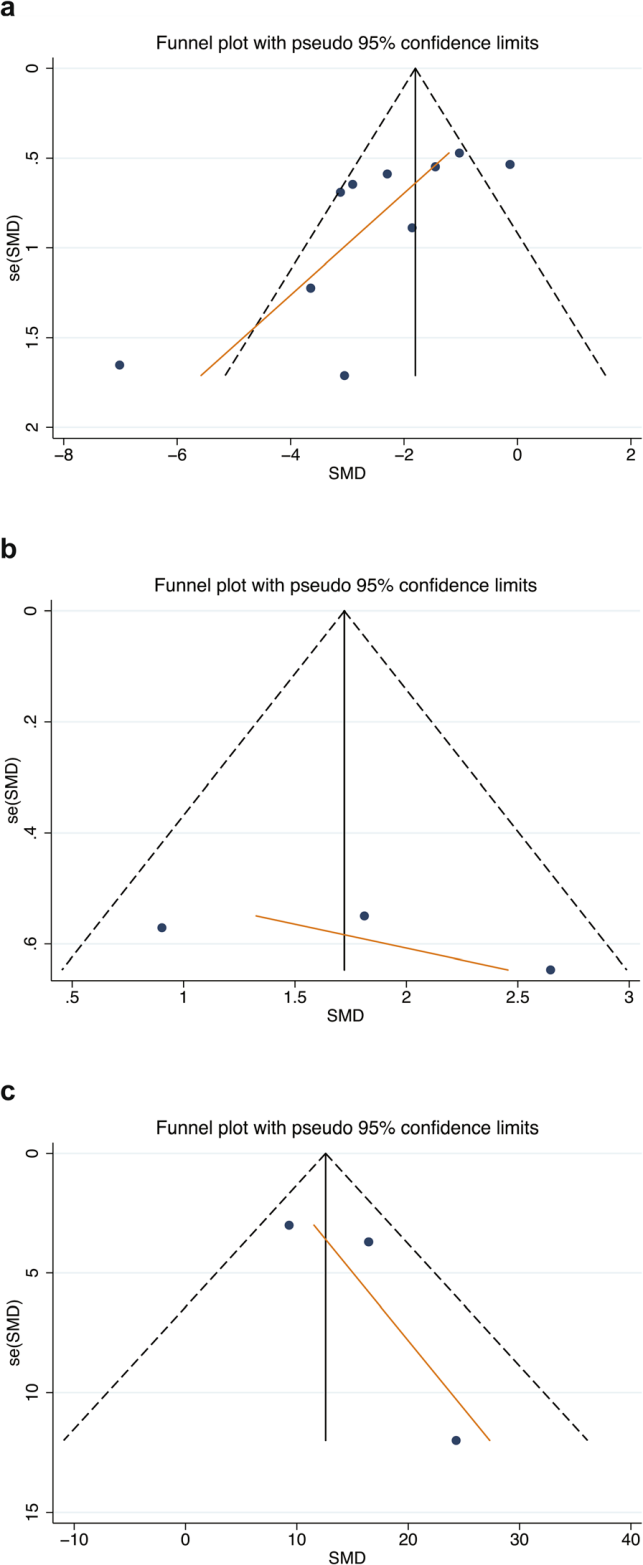
Funnel plot for the effect size of ADSC therapy. **a** Effect size of ADSC therapy on rotation behavior. **b** Effect size of ADSC therapy on rotarod test result. **c** Effect size of ADSC therapy on the number of TH-positive neurons. Dots represent individual studies. The solid vertical line represents the pooled effect size. The dashed diagonal lines represent pseudo-95% confidence limits around the pooled effect size for each standard error on the vertical axis. The diagonal solid line represents the fitted line between the treatment effect and its standard error for each study using Egger’s test

The funnel plots for rotation behavior (Fig. 6a) and TH-positive neurons (Fig. 6c) were asymmetric according to the Egger’s and Begg’s tests (Supplementary file 2). The funnel plot for rotarod was symmetric and *P* > 0.05 was obtained from both Egger’s and Begg’s tests (Fig. 6b). These findings implied the possibility of publication bias on rotation behavior, rotarod test and TH^+^ neurons in these studies.

## Discussion

The key pathology of PD includes the loss of A9 nigral neurons that provide dopaminergic innervation to the striatum [39] and the presence of α-synuclein-containing Lewy bodies in the brain [40]. Continuous loss of nigral DA neurons and the non-physiologic mode of drug delivery make stem cell therapy a promising strategy to alter the progression of this disease [41].

Autologous ADSCs circumvent ethical concerns and appear to be a suitable candidate of PD therapy [42]. This type of cells, which was first reported in 2001, is one of the most convenient and preferable types of stem cells [43]. ADSCs have been used in contexts of spinal cord injury-induced neuroinflammation [44], vascular remodeling [45], osteoarthritis [46], breast cancer [47], coronavirus disease 2019 [48], and neurodegenerative disorders [49–51].

As innovative preclinical assessment tools, neurotoxin-based PD animal models, which induce substantia nigra pars compacta dopaminergic neuronal death, can be used to assess therapeutic effects on PD symptoms and side effects associated with DA-replacement therapies [52, 53]. In this review, the included studies from 8 databases all used neurotoxin-based PD animal models, predominately the rodent 6-OHDA model, the first and classic animal model of PD [54]. All studies have shown the same direction of effect of ADSC treatment, suggesting that ADSCs are useful cell sources for treating PD. To evaluate the optimal forms of ADSCs, the experiments reporting rotation behavior were divided into two groups: those using primitive ADSCs and those using neural induction form of ADSCs. The neural-induction ADSCs were found to more effectively improve outcomes compared to the primitive ADSCs, suggesting that ADSCs, especially the neural-induction ADSCs, are potential therapeutic options for cell replacement therapy of PD. This finding is consistent with previous studies  in which stem cell therapies for PD tend to graft cells which are trans-differentiated into DA neurons as a direct supplement, in order to help reconstruct the nigrostriatal pathway [55–57]. Therefore, the ADSC-derived DA neurons provide an approach for PD treatment [42]. Due to the vulnerability of ADSCs after neural induction, intracerebral injection is the best route of administration [30]. Chi et al. have also documented that intracerebral injection of ADSCs could be applied to maximize the potential for recovery [31].

Then, we evaluated whether the follow-up time would influence the efficacy of ADSC treatment on rotation behavior, and investigated it as a source of heterogeneity in the data analysis. Subgroup analysis indicated significant ADSC treatment efficacy at either length of follow-up, suggesting that this therapy had a long-term effect. The heterogeneity of rotation data may be due to the route of administration, which varied among studies.

So what are the mechanisms underlying the functions of ADSCs? Based on these studies, the mechanisms involve neurogenesis and neuroprotection. DA neurons in the SN were increased after ADSC transplantation in all of the 10 studies, with quantification being done in seven studies. As for the neural-induction form of ADSCs, trans-differentiation and neurogenesis may be the main mechanisms of action. After transplantation of therapeutic ADSCs, the microenvironment is altered and neurotrophic factors are up-regulated, which provide a supplementary mechanism and nutritional niche in vivo [19, 25, 30].

Several studies have also reported that ADSCs do not adopt DA neuron fates *in vivo* [19, 24], and primitive ADSCs have more neuroprotective effects than the differentiated cell types.

## Conclusions

In conclusion, ADSC therapy is a promising regenerative therapeutic option for PD. The potential mechanisms underlying the effectiveness of ADSC therapy involve neurogenesis and neuroprotection. These findings imply that the successful application of ADSCs in clinical practice for PD relies on the neuronal induction form of ADSCs. However, larger studies are needed to confirm this conclusion.

## Supplementary Information

**Additional file 1:** Search strategy in Pubmed.

**Additional file 2:** **Table S1**. Inclusion criteria for experimental studies. **Table S2**. SYRCLE’s tool for assessing risk of bias. **Table S3**. List of the 10 studies that met the inclusion criteria based on full-text assessment. **Table S4**. Summary of study characteristics of the 10 studies that were included in the meta-analysis. **Table S5**. Risk of bias assessment for each included study

**Additional file 3:** Egger’s test and Begg’s test of publication bias.

## Data Availability

All relevant data are within the paper and the online Supplementary files.

## Abbreviations

PD: Parkinson’s disease
DA: Dopamine
SN: Substantia nigra
MSCs: Mesenchymal stem cells
ADSCs: Adipose-derived stem cells
6-OHDA: 6-hydroxydopamine
MPTP: 1-methyl-4-phenyl-1,2,3,6 tetrahydropyridine
TH: Tyrosine hydroxylase
BDNF: Brain-derived neurotrophic factor
IFN: Interferon
