# Supplementary material for: Effectiveness and mechanisms of adipose-derived stem cell therapy in animal models of Parkinson’s disease: a systematic review and meta-analysis
Source: Transl Neurodegener. 2021 Apr 29;10:14. doi: 10.1186/s40035-021-00238-1 (PMC8081767; doi:10.1186/s40035-021-00238-1)
Supplement: Supplementary file 1 — Additional file 1: Search strategy in Pubmed. [file 40035_2021_238_MOESM1_ESM.docx]

**Supplementary File 1. Search strategy in Pubmed**

(((("parkinson disease"[MeSH Terms] OR ("parkinson"[All Fields] AND "disease"[All Fields])) OR "parkinson disease"[All Fields]) OR (((("parkinson disease"[MeSH Terms] OR ("parkinson"[All Fields] AND "disease"[All Fields])) OR "parkinson disease"[All Fields]) OR ("parkinson s"[All Fields] AND "disease"[All Fields])) OR "parkinson s disease"[All Fields])) AND ((("mesenchymal stem cells"[MeSH Terms] OR (("mesenchymal"[All Fields] AND "stem"[All Fields]) AND "cells"[All Fields])) OR "mesenchymal stem cells"[All Fields]) OR (((("adipose"[All Fields] OR "adiposities"[All Fields]) OR "adiposity"[MeSH Terms]) OR "adiposity"[All Fields]) AND (((((((((((((((("analogs and derivatives"[MeSH Subheading] OR ("analogs"[All Fields] AND "derivatives"[All Fields])) OR "analogs and derivatives"[All Fields]) OR "derivatives"[All Fields]) OR "derivable"[All Fields]) OR "derivant"[All Fields]) OR "derivants"[All Fields]) OR "derivate"[All Fields]) OR "derivated"[All Fields]) OR "derivates"[All Fields]) OR "derivation"[All Fields]) OR "derivations"[All Fields]) OR "derivative"[All Fields]) OR "derive"[All Fields]) OR "derived"[All Fields]) OR "derives"[All Fields]) OR "deriving"[All Fields]) AND (("stem cells"[MeSH Terms] OR ("stem"[All Fields] AND "cells"[All Fields])) OR "stem cells"[All Fields])))) AND ("animals"[MeSH Terms:noexp] OR "animal"[All Fields])
