# Supplementary material for: Effectiveness and mechanisms of adipose-derived stem cell therapy in animal models of Parkinson’s disease: a systematic review and meta-analysis
Source: Transl Neurodegener. 2021 Apr 29;10:14. doi: 10.1186/s40035-021-00238-1 (PMC8081767; doi:10.1186/s40035-021-00238-1)
Supplement: Supplementary file 2 — Additional file 2: Table S1. Inclusion criteria for experimental studies. Table S2. SYRCLE’s tool for assessing risk of bias. Table S3. List of the 10 studies that met the inclusion criteria based on full-text assessment. Table S4. Summary of study characteristics of the 10 studies that were included in the meta-analysis. Table S5. Risk of bias assessment for each included study [file 40035_2021_238_MOESM2_ESM.docx]

**Table S1. Inclusion criteria for experimental studies**

| PICO | Specific inclusion |
| --- | --- |
| Population | 1. Animal models of Parkinson’s disease |
| Interventions | 1. Adipose-derived stem cells intervention |
| Comparators | 1. Prospective controlled experiments using animals |
|  | 1. Marched control group of animals with induced Parkinson’s disease with receive control treatment or without any intervention |
| Outcomes | 1. Immunohistochemical testing and/or mRNA expression changes to reveal the mechanisms |
|  | 1. Effects on clinical outcome defined as behaviors testing like rotation, 8-arm maze, rotarod |

**Table S2.** **SYRCLE’s tool for assessing risk of bias**

| Item | Domain | Type of bias |
| --- | --- | --- |
| Q1. Was the allocation sequence adequately generated and applied? | Sequence generation | Selection bias |
| Q2. Were the groups similar at baseline or were they adjusted for confounders in the analysis? | Baseline characteristics | Selection bias |
| Q3. Was the allocation to the different groups adequately concealed during? | Allocation concealment | Selection bias |
| Q4. Were the animals randomly housed during the experiment? | Random housing | Performance bias |
| Q5. Were the caregivers and/or investigators blinded from knowledge which intervention each animal received during the experiment? | Blinding | Performance bias |
| Q6. Were animals selected at random for outcome assessment? | Random outcome assessment | Detection bias |
| Q7. Was the outcome assessor blinded? | Blinding | Detection bias |
| Q8. Were incomplete outcome data adequately addressed? | Incomplete outcome data | Attrition bias |
| Q9. Are reports of the study free of selective outcome reporting? | Selective outcome reporting | Reporting bias |
| Q10. Was the study apparently free of other problems that could result in high risk of bias? | Other sources of bias | Other |

**Table S3.** **List of the 10 studies that met the inclusion criteria based on full-text assessment**

| Author(year) | Type of intervention | Model | Species/Strains | Groups | Route administration | Doses per animal | Time between lesion and treatment | Follow-up time | Outcome parameters | Results | Mechanisms |
| --- | --- | --- | --- | --- | --- | --- | --- | --- | --- | --- | --- |
| McCoy(2008) | Autograft; Neural induction;  Expanded P2-P4 | 6-OHDA | Rats/Sprague-Dawley | Neural=5; Naïve=5; Saline=4; Unlesioned=4 | Intrastriatal | 4$\times$10^4^ | 1 week | 4 weeks | Rotation(turns/20min);Number of TH^+^ neurons in SNpc and striatum(cells); Microglial burden in SNpc(IOD) | Behavior improvement; Neuroprotective effects of both ADSCs grafts | Exert trophic factors at the lesion site; Attenuate microglial activation |
| Zhou(2013) | Autograft; Neural induction | MPTP(hemi) | Monkeys/Rhesus | Neuronal-primed ADSCs and Ad-NTN-TH=3; Ad-NTN-TH=3;Neuronal-primed ADSCs=3; HBSS=3 | Intrastriatal | 6$\times$10^6^ | 12 months | 4 months | UPDRS(scores); Rotation(turns/min); Percentage of TH^+^ neurons(%) in substantial nigra | Neuronal-primed ADSCs alone don’t survive the brain significantly |  |
| Park(2014) | Xenograft; Original;  Expanded 5 days | 6-OHDA | Rats/Sprague-Dawley | Low density(LD)-ADSCs=8; High density(HD)- ADSCs=11;  HBSS=10 | Cisterna magna injection | 2$\times$10^6^ | 1 week | 6 weeks | Rotation(turns/50min); Relative neuronal protein expression of midbrain tissues | HD-ADSCs have behavior improvement | BDNF expression and subsequent increase of proliferation in neuronal cells |
| Berg(2015) | Xenograft; Original;  Expanded P4(aMSC);  Expanded  3-4 days(sMSC) | 6-OHDA | Rats/Wistar | Adherent ADSCs(aADSCs)=10; Spheroid ADSCs(sADSCs)=10; 6-OHDA=11; Sham=16 | Intranigral | 3$\times$10^5^ | 1 week | 3 weeks | 8-arm radial maze(times); Rotation(turns/min); Number of newly generated cells in the adult DG(cells);Nigral mRNA levels of GDNF, BDNF and GFAP; Microglial in transplantation area(cells) | aADSCs sifnificantly increase TH and BDNF levels in the SN, improve motor functions; sADSCs support nigral neurodegeneration, increase local inflammation and suppress hippocampal neurogenesis | Paracrine effects of the graft; Increasing numbers of GFAP levels; An increased local inflammatory response to the graft could lead to a suppressed neuroprotective effect |
| Choi(2015) | Xenograft; Original;  Expanded P3;  Cell line (RNL Bio) | 6-OHDA | Mice/C57BL6 | 6-OHDA/hADSCs=5; 6-OHDA=5; Saline/hADSCs=5; Saline=5 | Intravenous | 1$\times$10^6^; Twice at an interval of 2 weeks | 3 weeks | 6 weeks | Rotation(turns/30min); Rotarod(sec); Number of TH Neurons in the SN(cells); PET imaging analysis of dopamine D2 receptor in the striatum(binding potential); The population of damaged mitochondria(%); Mitochondrial complex I activity(%) | Motor improved; Rescue dopaminergic neurons death; Decrease the ruptured and damaged mitochondria in the SN; Restore the decreased mitochondrial complex I activity | The restored dopaminergic cell death could be explained by recovering the mitochondrial dysfunction |
| Schwerk(2015) | Xenograft; Original;  Expanded after P2 | 6-OHDA | Rats/Wistar | 6-OHDA/hADSCs=7; 6-OHDA/NaCl=7; Sham/NaCl=7; | Intranigral | 3$\times$10^5^ | 1 week | 6 months | Rotation(turns/min); 8-arm radial maze(times); Percentage of TH^+^ neurons in the SN(%); Neurogenesis of subventricular and hippocampal cells(cells); EPO, IL-10, IL-4, IL-2(pg/ml); | Improve working memory; Reduce dopaminergic degeneration in the SN | Transplanted ADSCs alter cytokines levels and growth factors to induce neuroprotection with the local microenvironment; Generation of newborn neurons; Anti-apoptotic and anti-inflammatory |
| Takahashi(2017) | Xenograft; Neural induction;  CD31-negative cells isolated | 6-OHDA | Rats/Wistar | 6-OHDA/ADSCs=12; 6-OHDA/Hank’s solution=10 | Ipsilateral MFB injection | 4$\times$10^5^ | Unclear | 4 weeks | Rotation(turns/min) | Improve the symptoms | Transplanted cells generate DA neurons in vivo |
| Chi(2018) | Xenograft; BP treated/Original | MPTP | Mice/C57BL6 | Pretreat intracerebral(i.c.) i.c.+intravenous(i.v.)=6; ADSC i.c.=7; Pretreat i.c. =7; Saline=7; Control=6;Pretreat i.c. EtOH=3(day22) | Intrastriatal and/or Intravenous | 1$\times$10^6^ | 2 days or 4days(i.v.) | 3 weeks | Beam walking(sec); Rotarod(sec); Locomotor activity(mm); Comparable TH quantity of the SN(%); | Improve the behavior; Increase the numbers of TH-expressing cells | BP stimulates neurogenesis in ADSCs |
| Meligy(2019) | Xenograft; Original | Rotenone | Rats/Wistar | PD=10; PD-ADSCs=10; PD-carbidopa/ levodopa=10; Control=10 | Intracardiac | 1$\times$10^6^ | 2 weeks | 2 weeks | Rotarod(sec); Activity cage(count/5min); Pole test(sec); Pale and dark neurons in SNc(cells); TH^+^ cells in the SNc(cells/mm^2^); Blood levels of angiopoietin-2(ng/mL) and dopamine(pg/mL); GFAP and Nestin mRNA gene expression | Improve the behavior; TH^+^ neurons increase | ADSCs have a regenerative effect; ADSCs can induct angiogenesis, reduce the GFAP and Nestin gene expression in peripheral blood |
| Moayeri(2020) | Allograft; Original;  Expanded after P4 | 6-HD | Rats/Sprague-Dawley | PD=7; Sham=7; PD/ADSC=7; PD/ADSC/SPION=7; PD/ADSC/SPION/EM=7 | Ipsilateral MFB injection | 3$\times$10^5^ | 2 weeks | 6 weeks | Rotation(turns/1h); Nissl stained cell in the SNc(cells) | Improve the behavior; Increase the dopaminergic neurons | Homing of stem cells in the target tissue |

Abbreviations: TH, tyrosine hydroxylase; SNc, substantia nigra compacta; DG, dentate gyrus; MFB, medial forebrain bundle; BP, n-butylidenephthali

**Table S4. Summary of study characteristics of the 10 studies that were included in the meta-analysis.**

| **Study characteristic** | **Sub-groups** | **Numbers of studies** |
| --- | --- | --- |
| **Model of PD** | 6-OHDA | 7 |
|  | MPTP | 2 |
|  | Rotenone | 1 |
| **Species** | Rats | 7 |
|  | Mice | 2 |
|  | Monkeys | 1 |
| **Type of intervention** | ADSCs | 7 |
|  | Neural differentiated ADSCs | 3 |
| **Route of administration** | Intracerebral（Intrastriatal/Intranigral/Ipsilateral MFB/Cisterna magna） | 8(3/2/2/1) |
|  | Intravenous | 1 |
|  | Intracardiac | 1 |
| **ADSCs doses** | <1$\times$10^6^ | 5 |
|  | $\geq$1$\times$10^6^ | 5 |
| **Time between lesion and treatment** | $\leq$1 week | 5 |
|  | >1 week | 4 |
|  | Unclear | 1 |
| **Duration of follow-up period** | $\leq$4 weeks | 5 |
|  | >4 weeks | 5 |
| **Behavior outcomes** | Rotation | 8 |
|  | Rotarod | 3 |
|  | 8-arm maze | 2 |
|  | Locomotor activity | 1 |
|  | Pole test | 1 |
|  | Beam walking | 1 |
|  | UPDRS | 1 |
|  | Activity cage | 1 |
| **Main in vivo changes** | TH^+^ neurons | 7 |

**Table S5. Risk of bias assessment for each included study**

|  | Q1 | Q2 | Q3 | Q4 | Q5 | Q6 | Q7 | Q8 | Q9 | Q10 |
| --- | --- | --- | --- | --- | --- | --- | --- | --- | --- | --- |
| **McCoy 2008** | U | N | U | U/Y | U | U | U/Y | U | N | Y/Y/Y/U |
| **Zhou 2013** | Y | Y | U | U/Y | U | U | U/N | Y | Y | Y/Y/N/U |
| **Park 2014** | U | Y | U | U/Y | U | U | U/Y | U | U | Y/Y/Y/U |
| **Berg 2015** | U | Y | U | U/Y | U | U | U/Y | Y | N | Y/Y/Y/U |
| **Choi 2015** | U | U | U | U/Y | U | U | U/Y | U | Y | Y/Y/N/U |
| **Schwerk 2015** | U | N | U | U/Y | U | U | U/Y | U | N | Y/Y/Y/U |
| **Takahashi 2017** | U | U | U | U/Y | U | U | U/Y | U | Y | Y/Y/Y/N |
| **Chi 2018** | U | Y | U | U/Y | U | U | U/Y | U | Y | Y/Y/Y/U |
| **Meligy 2019** | U | Y | U | U/Y | U | U | U/Y | Y | Y | Y/Y/Y/U |
| **Moayeri 2020** | U | Y | U | U/Y | U | U | U/Y | U | N | Y/Y/N/U |

Abbreviations: Y, yes; N, no; U, unclear
