# Supplementary material for: Effectiveness and mechanisms of adipose-derived stem cell therapy in animal models of Parkinson’s disease: a systematic review and meta-analysis
Source: Transl Neurodegener. 2021 Apr 29;10:14. doi: 10.1186/s40035-021-00238-1 (PMC8081767; doi:10.1186/s40035-021-00238-1)
Supplement: Supplementary file 3 — Additional file 3: Egger’s test and Begg’s test of publication bias. [file 40035_2021_238_MOESM3_ESM.docx]

**Supplementary File 3. Egger’s test and Begg’s test of publication bias**

**Rotation：**

Begg's Test

adj. Kendall's Score (P-Q) = -23

Std. Dev. of Score = 11.18

Number of Studies = 10

z = -2.06

Pr > |z| = 0.040

z = 1.97 (continuity corrected)

Pr > |z| = 0.049 (continuity corrected)

Egger's Test

----------------------------------------------------------------------------------------------------------------------

Std_Eff | Coef. Std. Err. t P>|t| [95% Conf. Interval]

----------------------------------------------------------------------------------------------------------------------

slope | 1.377714 1.256619 1.10 0.305 -1.520056 4.275483

bias | -5.046051 2.183208 -2.31 0.050 -10.08054 -.011564

----------------------------------------------------------------------------------------------------------------------

**Rotarod：**

Begg's Test

adj. Kendall's Score (P-Q) = 1

Std. Dev. of Score = 1.91

Number of Studies = 3

z = 0.52

Pr > |z| = 0.602

z = 0.00 (continuity corrected)

Pr > |z| = 1.000 (continuity corrected)

Egger's Test

----------------------------------------------------------------------------------------------------------------------

Std_Eff | Coef. Std. Err. t P>|t| [95% Conf. Interval]

----------------------------------------------------------------------------------------------------------------------

slope | -4.702451 8.517449 -0.55 0.679 -112.9269 103.522

bias | 11.20324 14.75227 0.76 0.587 -176.2422 198.6487

----------------------------------------------------------------------------------------------------------------------

**TH：**

Begg's Test

adj. Kendall's Score (P-Q) = 1

Std. Dev. of Score = 1.91

Number of Studies = 3

z = 0.52

Pr > |z| = 0.602

z = 0.00 (continuity corrected)

Pr > |z| = 1.000 (continuity corrected)

Egger's Test

----------------------------------------------------------------------------------------------------------------------

Std_Eff | Coef. Std. Err. t P>|t| [95% Conf. Interval] ----------------------------------------------------------------------------------------------------------------------

slope | -2.540808 13.4306 -0.19 0.881 -173.1927 168.1111

bias | 4.334134 4.086198 1.06 0.481 -47.58594 56.25421

----------------------------------------------------------------------------------------------------------------------
